# Supplementary material for: Shared community effects and the non-genetic maternal environment shape cortisol levels in wild chimpanzees
Source: Commun Biol. 2023 May 26;6:565. doi: 10.1038/s42003-023-04909-9 (PMC10214342; doi:10.1038/s42003-023-04909-9)
Supplement: Supplementary file 2 — Supplementary Material [file 42003_2023_4909_MOESM2_ESM.pdf]

# Shared community effects and the non-genetic maternal environment shape cortisol levels in wild chimpanzees : Supplementary Materials

Patrick J. Tkaczynski, Fabrizio Mafessoni, Cédric Girard-Buttoz, Liran Samuni, Corinne Y. Ackermann, Pawel Fedurek, Cristina Gomes, Catherine Hobaiter, Therese Löhrich, Virgile Manin, Anna Preis, Prince D. Valé, Erin G. Wessling, Livia Wittiger, Zinta Zommers, Klaus Zuberbuehler, Linda Vigilant, Tobias Deschner, Roman M. Wittig, Catherine Crockford

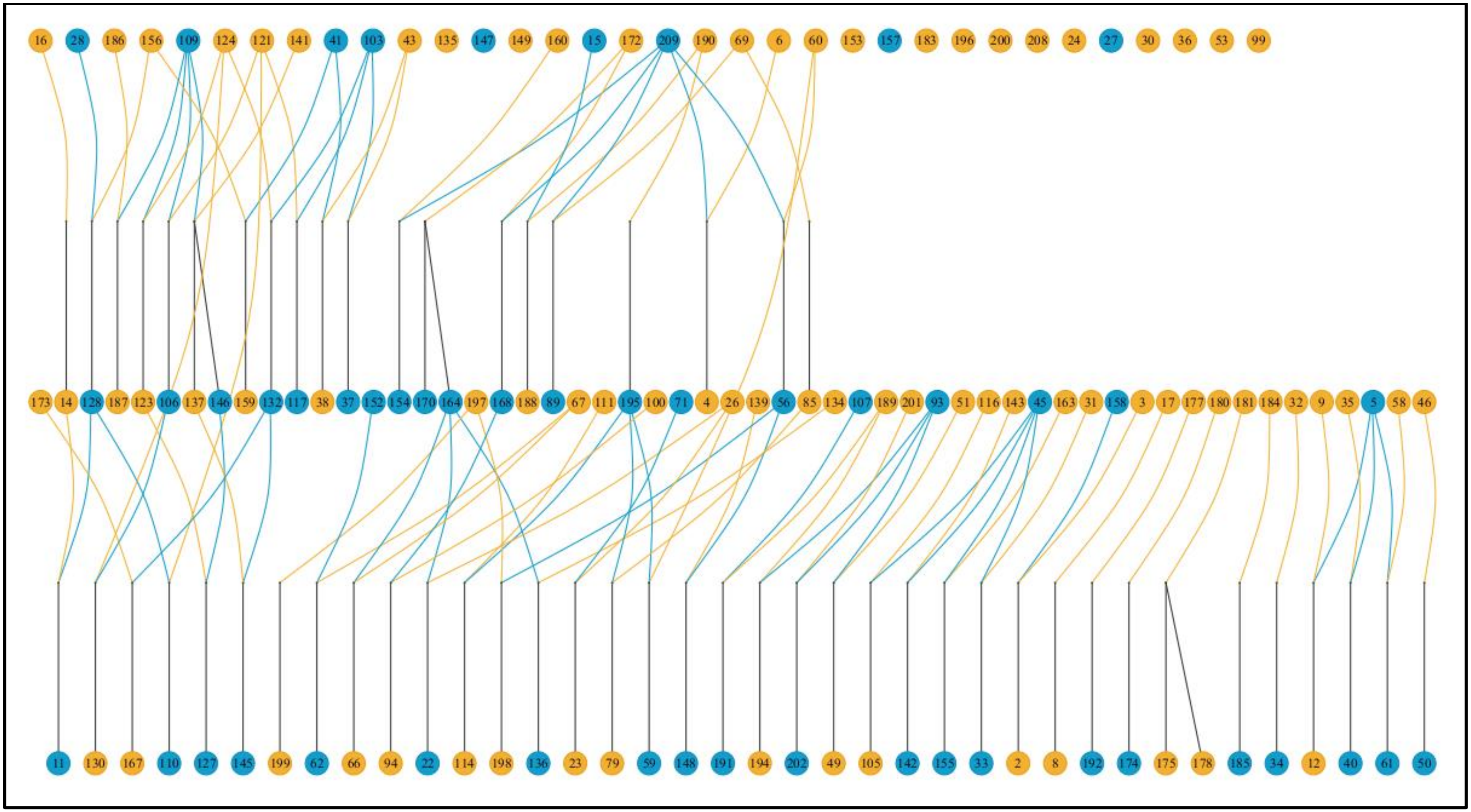

Figure S1: Pedigree of individuals from the Taï field site included within our analysis. Males are represented by the blue circles, females by the yellow circles. Note, this pedigree illustration includes the mothers and/or fathers of individuals with urinary cortisol values in our study, even if these mothers and/fathers were not sampled themselves. However, the offspring of individuals are only included if the offspring themselves had urinary cortisol values in the study.

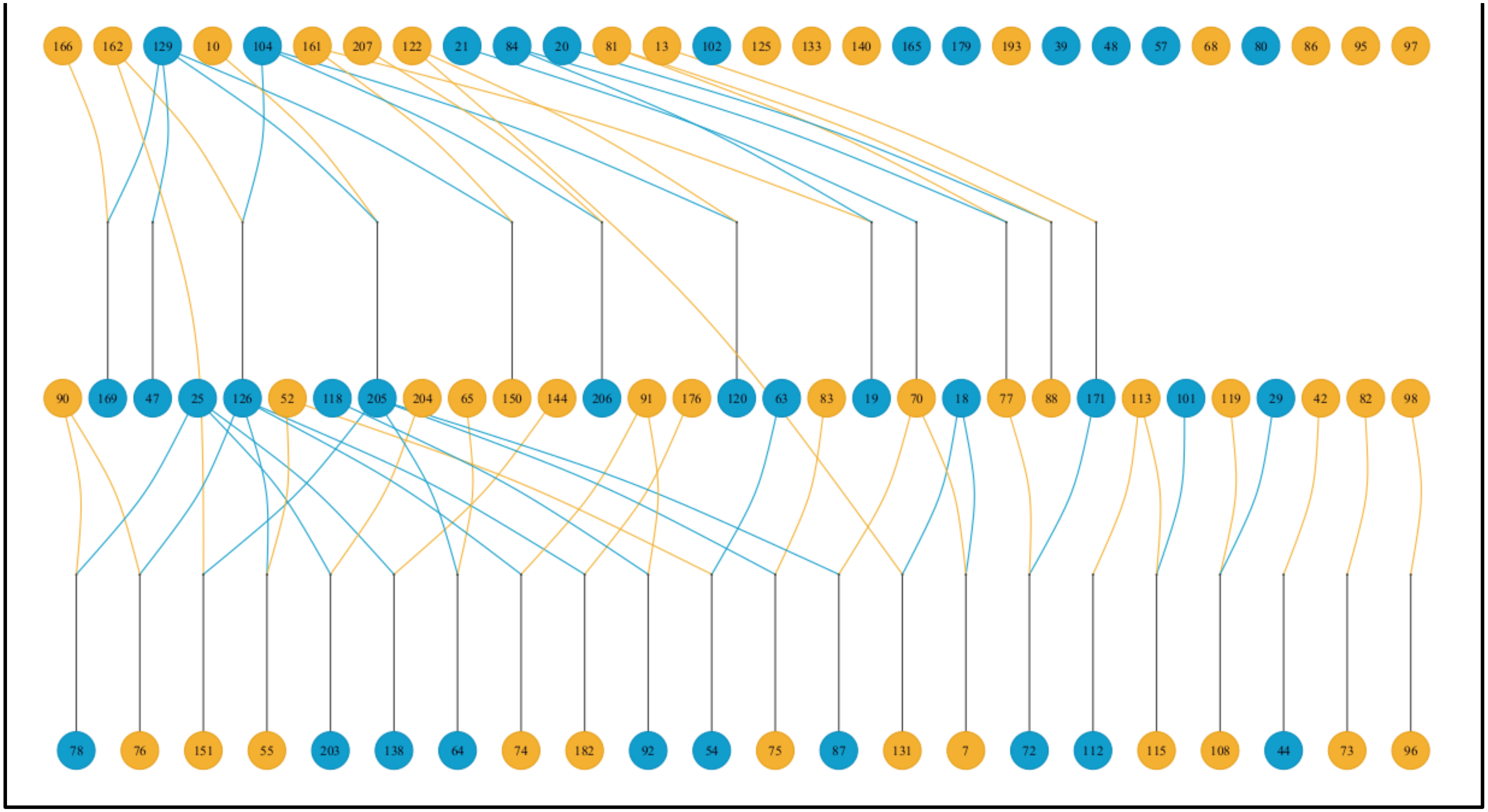

Figure S2: Pedigree of individuals from the Budongo field site included within our analysis. Males are represented by the blue circles, females by the yellow circles. Note, this pedigree illustration includes the mothers and/or fathers of individuals with urinary cortisol values in our study, even if these mothers and/fathers were not sampled themselves. However, the offspring of individuals are only included if the offspring themselves had urinary cortisol values in the study.

Table S1: Model comparison results using leave-one-out cross validation and the loo_compare function of the 'loo' R package. Model comparisons were conducted for all individuals, then for models built for adult males, adult females, and juvenile individuals separately. In each case, the model with the strongest support is highlighted in bold.

**Demographic**	**Model**	**Expected logwise predictive density**	**Standard error**
All individuals	**Random intercept**	**0.000**	**0.000**
	Reaction norm	-3.040	4.483
	Null model	-376.724	28.632
Adult males	**Random intercept**	**0.000**	**0.000**
	Reaction norm	-0.144	3.605
	Null model	-92.164	13.979
Adult females	**Random intercept**	0.000	0.000
	Reaction norm	-1.655	2.512
	Null model	-61.496	11.692
Juveniles	**Reaction norm**	**0.000**	**0.000**
	Random intercept	-14.197	7.832
	Null model	-62.995	13.791

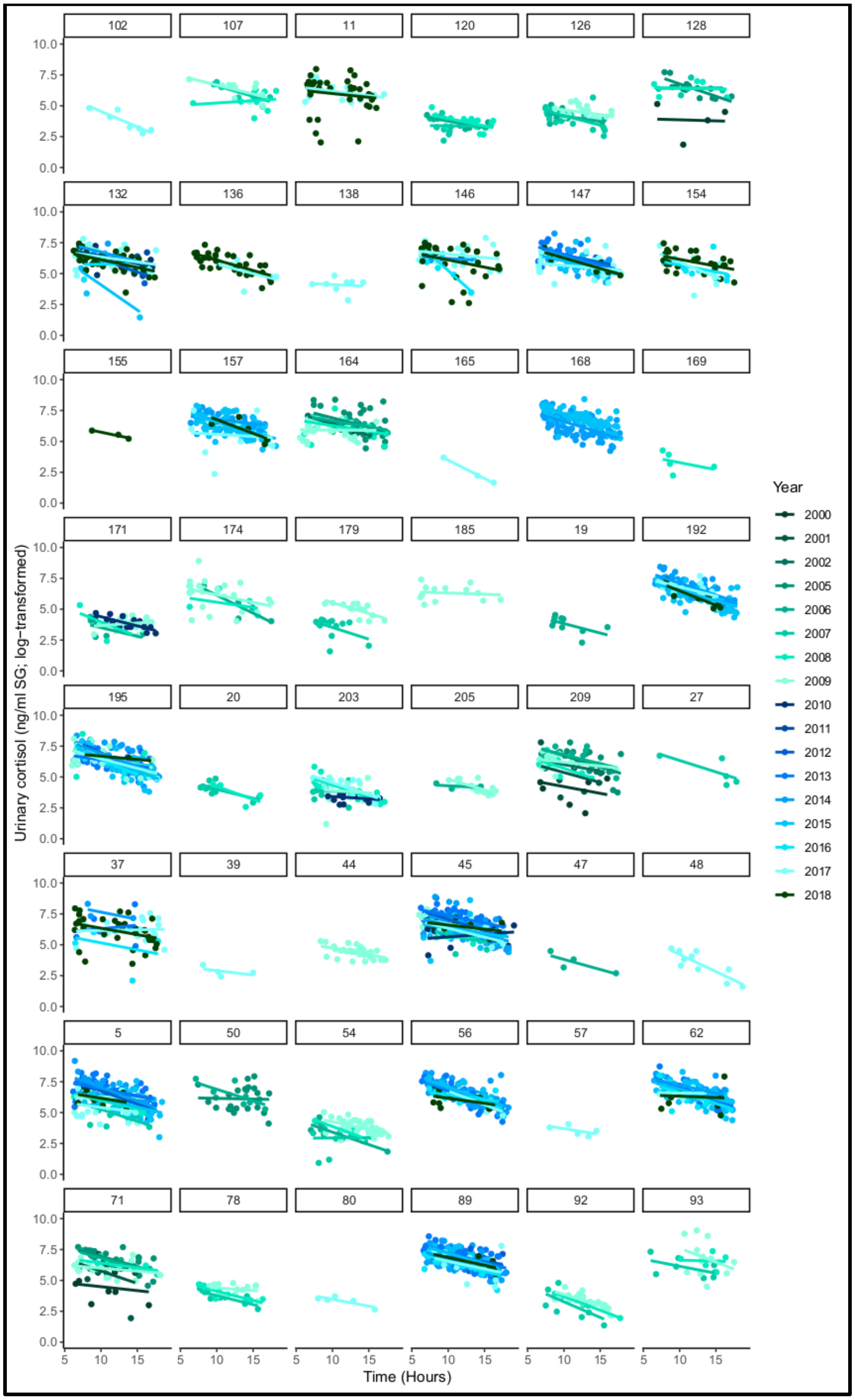

Figure S3: Urinary cortisol concentration (ng/ml SG; log transformed) circadian reaction norms for all individuals when appearing as adult males in the study (n =48). The points represent individual sample values, the slopes individual responses to time of day; both sample values and responses are shaded according to the year in which they were collected respectively. The numbers above panels indicate individual identity as it appears in the pedigree (Figures S1 and S2).

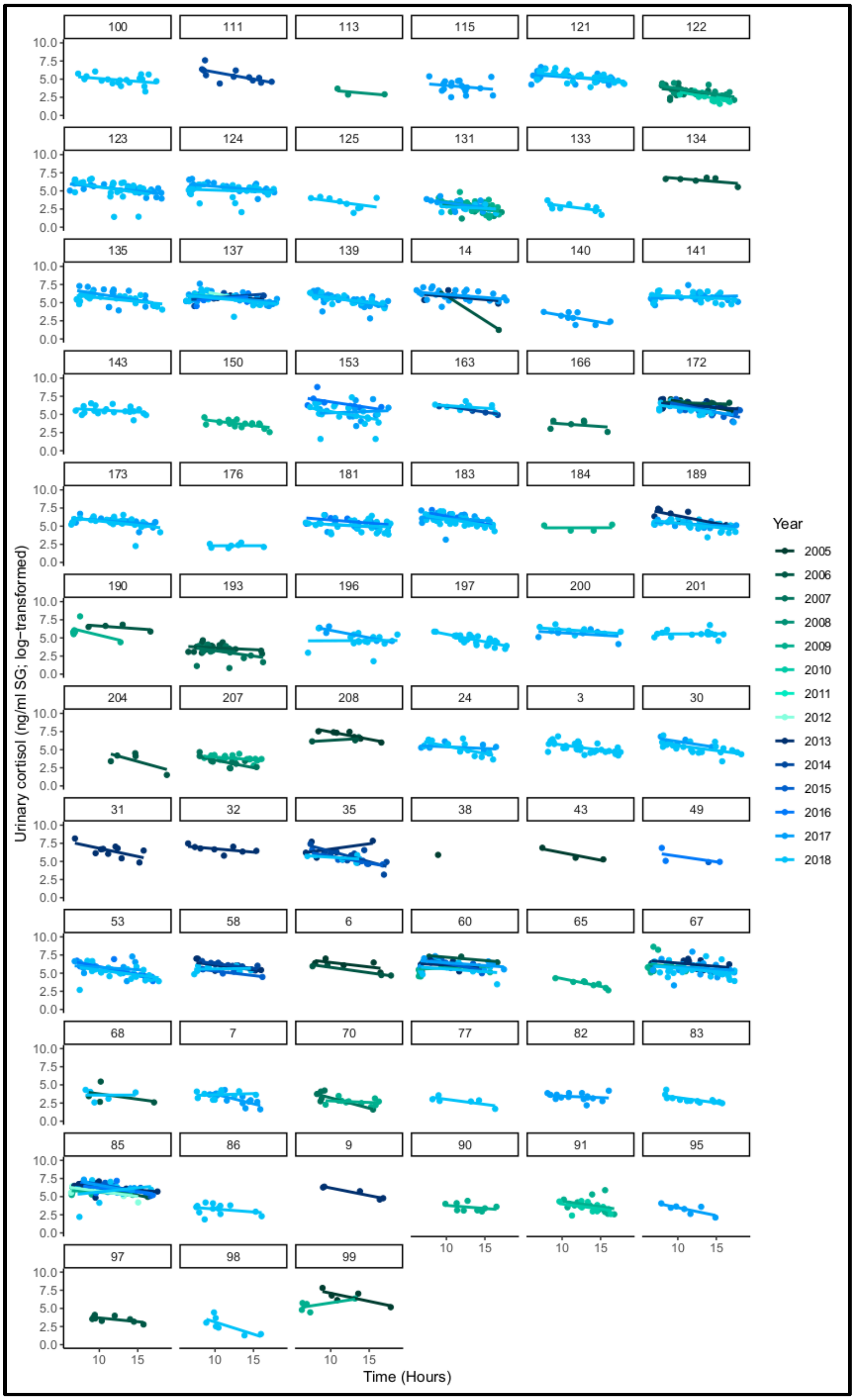

Figure S4: Urinary cortisol concentration (ng/ml SG; log transformed) circadian reaction norms for all individuals when appearing as adult females in the study (n =69). The points represent individual sample values, the slopes individual responses to time of day; both sample values and responses are shaded according to the year in which they were collected respectively. The numbers above panels indicate individual identity as it appears in the pedigree (Figures S1 and S2). Individual 38 transitioned into adulthood during one year of sampling and, therefore, has only sample appearing in this plot (see Figure S5).

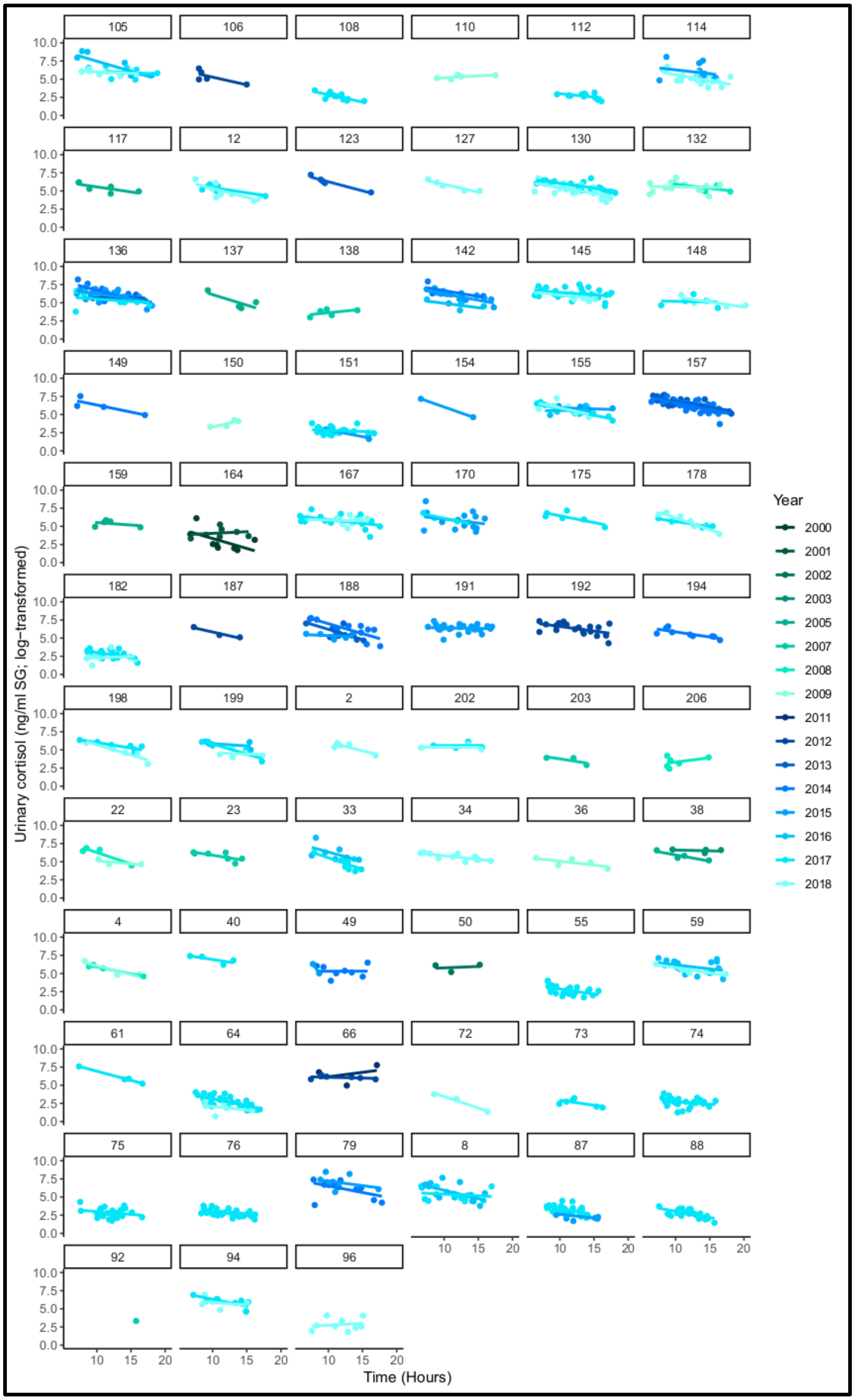

Figure S5: Urinary cortisol concentration (ng/ml SG; log transformed) circadian reaction norms for all individuals when appearing as immature individuals in the study (n = 69). The points represent individual sample values, the slopes individual responses to time of day; both sample values and responses are shaded according to the year in which they were collected respectively. The numbers above panels indicate individual identity as it appears in the pedigree (Figures S1 and S2). Individual 92 transitioned into adulthood during one year of sampling and, therefore, has only one sample appearing in this plot (see Figure S3).

Table S2: Fixed effect results from the reaction norm LMM estimating urinary cortisol concentrations (ng/ml SG) in response to time of day across ***all chimpanzees*** *in five chimpanzee communities. Categorical variables have the reference category in parentheses. Effects where 95% credible intervals do not cross 0 are indicated in bold.*

**Variable**	**Estimate**	**Est. error**	**Q2.5**	**Q97.5**
Intercept	4.989	0.454	3.980	5.851
**Time of day^2**	**-0.175**	**0.071**	**-0.315**	**-0.035**
**Demographic (adult males)**	**-0.371**	**0.052**	**-0.473**	**-0.269**
**Time of day**	**-0.715**	**0.033**	**-0.781**	**-0.649**
Age at sample	0.118	0.056	0.010	0.228
**Sin date**	**-0.130**	**0.021**	**-0.173**	**-0.089**
Cos date	0.123	0.022	0.081	0.167
Sex ratio	0.122	0.175	-0.217	0.475
**Community size**	**-1.353**	**0.477**	**-2.177**	**-0.309**
LCMS code (new method)	0.313	0.143	0.036	0.599
Time of day^2 : Demographic	-0.026	0.098	-0.218	0.165
Time of day : Demographic	0.020	0.050	-0.076	0.117
**Time of day^2 : Age at sample**	**-0.278**	**0.086**	**-0.450**	**-0.110**
Time of day : Age at sample	0.146	0.042	0.065	0.230
Time of day^2 : Sin date	0.036	0.057	-0.077	0.148
**Time of day: Sin date**	**-0.091**	**0.029**	**-0.149**	**-0.035**
Time of day^2: Cos date	0.156	0.058	0.044	0.270
Time of day: Cos date	-0.127	0.028	-0.182	-0.071
Time of day^2 : Sex ratio	-0.174	0.100	-0.368	0.026
Time of day : Sex ratio	0.055	0.046	-0.034	0.144
Time of day^2 : Community size	0.164	0.112	-0.057	0.381
Time of day : Community size	-0.063	0.051	-0.165	0.038
Time of day^2 : LCMS code (new method)	-0.121	0.101	-0.318	0.082
Time of day : LCMS code (new method)	-0.071	0.052	-0.172	0.033

Table S3: Random effect results from the reaction norm LMM estimating urinary cortisol concentrations (ng/ml SG) in response to time of day in five chimpanzee communities.

	Name	Variance	Est.error	Q2.5	Q97.5
Group		0.86	0.58	0.26	2.41
Project identity		0.31	0.09	0.17	0.53
Group-year		0.51	0.07	0.38	0.67
ID-year	Intercept	0.23	0.03	0.18	0.28
	Time of day^2	0.16	0.10	0.01	0.37
	Time of day	0.13	0.05	0.02	0.22
Individual identity	Intercept	0.24	0.03	0.18	0.30
	Time of day^2	0.07	0.06	0.00	0.22
	Time of day	0.04	0.03	0.00	0.11
Residual		0.65	0.01	0.64	0.67

Table S4: Fixed effect results from the reaction norm LMM estimating urinary cortisol concentrations (ng/ml SG) in response to time of day among ***adult males*** *in five chimpanzee communities. Categorical variables have the reference category in parentheses. Effects where 95% credible intervals do not cross 0 are indicated in bold.*

**Variable**	**Estimate**	**Est. error**	**Q2.5**	**Q97.5**
Intercept	5.556	0.442	4.563	6.380
Time of day^2	-0.192	0.128	-0.445	0.062
**Rank**	**0.288**	**0.074**	**0.138**	**0.430**
**Time of day**	**-0.685**	**0.063**	**-0.812**	**-0.566**
Age at sample	0.141	0.080	-0.008	0.305
**Sin date**	**-0.150**	**0.031**	**-0.212**	**-0.090**
**Cos date**	**0.096**	**0.032**	**0.033**	**0.160**
Sex ratio	-0.008	0.160	-0.334	0.302
Community size	-0.905	0.453	-1.698	0.164
LCMS code (new method)	0.101	0.171	-0.218	0.444
Time of day^2 : Rank	0.053	0.167	-0.280	0.387
Time of day : Rank	0.023	0.079	-0.126	0.182
Time of day^2 : Age at sample	-0.131	0.125	-0.377	0.111
Time of day : Age at sample	0.127	0.066	-0.004	0.257
Time of day^2 : Sin date	0.037	0.084	-0.129	0.203
**Time of day: Sin date**	**-0.151**	**0.040**	**-0.225**	**-0.072**
**Time of day^2: Cos date**	**0.267**	**0.089**	**0.096**	**0.447**
**Time of day: Cos date**	**-0.176**	**0.041**	**-0.258**	**-0.094**
Time of day^2 : Sex ratio	-0.181	0.132	-0.439	0.071
Time of day : Sex ratio	0.051	0.063	-0.076	0.174
Time of day^2 : Community size	0.239	0.190	-0.134	0.617
Time of day : Community size	-0.061	0.093	-0.244	0.123
Time of day^2 : LCMS code (new method)	-0.074	0.150	-0.367	0.233
Time of day : LCMS code (new method)	-0.111	0.074	-0.255	0.037

Table S5: Random effect results from the reaction norm LMM estimating urinary cortisol concentrations (ng/ml SG) in response to time of day among ***adult males*** *in five chimpanzee communities.*

	Name	Variance	Est.error	Q2.5	Q97.5
Group		0.76	0.57	0.14	2.28
Project identity		0.23	0.08	0.11	0.43
Group-year		0.57	0.08	0.42	0.75
ID-year	Intercept	0.21	0.04	0.14	0.29
	Time of day^2	0.19	0.12	0.01	0.44
	Time of day	0.13	0.06	0.01	0.24
Individual identity	Intercept	0.17	0.05	0.07	0.27
	Time of day^2	0.08	0.06	0.00	0.24
	Time of day	0.06	0.04	0.00	0.16
Residual		0.66	0.01	0.64	0.68

Table S6: Fixed effect results from the reaction norm LMM estimating urinary cortisol concentrations (ng/ml SG) in response to time of day among ***adult females*** *in five chimpanzee communities. Categorical variables have the reference category in parentheses. Effects where 95% credible intervals do not cross 0 are indicated in bold.*

Variable	Estimate	Est. error	Q2.5	Q97.5
Intercept	4.883	0.614	3.561	6.022
Time of day^2	-0.132	0.274	-0.677	0.402
Reproductive state (cycling)	-0.108	0.124	-0.353	0.141
**Time of day**	**-0.547**	**0.137**	**-0.818**	**-0.276**
Age at sample	0.181	0.097	-0.009	0.372
**Sin date**	**-0.148**	**0.040**	**-0.225**	**-0.071**
**Cos date**	**0.180**	**0.042**	**0.099**	**0.262**
Sex ratio	-0.233	0.185	-0.605	0.126
Community size	-0.654	0.645	-1.810	0.661
**LCMS code (new method)**	**0.502**	**0.195**	**0.135**	**0.899**
Time of day^2 : Reproductive state (cycling)	-0.227	0.279	-0.774	0.319
Time of day : Reproductive state (cycling)	-0.166	0.138	-0.436	0.112
Time of day^2 : Age at sample	-0.030	0.176	-0.366	0.316
**Time of day : Age at sample**	**0.163**	**0.081**	**0.004**	**0.320**
Time of day^2 : Sin date	0.158	0.113	-0.061	0.381
Time of day: Sin date	-0.031	0.052	-0.132	0.072
Time of day^2: Cos date	0.030	0.110	-0.191	0.237
Time of day: Cos date	-0.070	0.052	-0.169	0.029
Time of day^2 : Sex ratio	0.065	0.227	-0.387	0.504
Time of day : Sex ratio	0.145	0.099	-0.044	0.345
Time of day^2 : Community size	0.268	0.250	-0.214	0.762
Time of day : Community size	-0.199	0.110	-0.409	0.020
**Time of day^2 : LCMS code (new method)**	**-0.438**	**0.216**	**-0.862**	**-0.014**
Time of day : LCMS code (new method)	-0.040	0.109	-0.256	0.172

*Table S7: Random effect results from the reaction norm LMM estimating urinary cortisol concentrations (ng/ml SG) in response to time of day among **adult females** in five chimpanzee communities.*

	Name	Variance	Est.error	Q2.5	Q97.5
Group		1.18	0.72	0.32	2.98
Project identity		0.29	0.11	0.14	0.56
Group-year		0.15	0.06	0.04	0.27
ID-year	Intercept	0.09	0.05	0.00	0.20
	Time of day^2	0.23	0.13	0.01	0.48
	Time of day	0.08	0.06	0.00	0.21
Individual identity	Intercept	0.26	0.05	0.17	0.35
	Time of day^2	0.18	0.11	0.01	0.41
	Time of day	0.07	0.05	0.00	0.18
Residual		0.66	0.01	0.63	0.68

*Table S8: Fixed effect results from the reaction norm LMM estimating urinary cortisol concentrations (ng/ml SG) in response to time of day among **immatures** in five chimpanzee communities. Categorical variables have the reference category in parentheses. Effects where 95% credible intervals do not cross 0 are indicated in bold.*

Variable	Estimate	Est. error	Q2.5	Q97.5
Intercept	4.700	0.452	3.716	5.537
Time of day^2	0.093	0.160	-0.219	0.412
Sex (female)	-0.046	0.129	-0.306	0.208
**Time of day**	**-0.829**	**0.077**	**-0.980**	**-0.674**
**Age at sample**	**-0.325**	**0.127**	**-0.578**	**-0.086**
Sin date	-0.064	0.047	-0.159	0.027
Cos date	0.051	0.048	-0.045	0.141
Sex ratio	0.149	0.169	-0.169	0.493
**Community size**	**-1.702**	**0.672**	**-2.769**	**-0.122**
LCMS code (new method)	0.094	0.278	-0.440	0.645
Time of day^2 : Sex (female)	0.043	0.226	-0.390	0.485
Time of day : Sex (female)	0.014	0.115	-0.219	0.243
Time of day^2 : Age at sample	0.237	0.227	-0.206	0.687
Time of day : Age at sample	-0.021	0.116	-0.243	0.211
Time of day^2 : Sin date	0.012	0.138	-0.249	0.289
Time of day: Sin date	0.080	0.069	-0.055	0.214
Time of day^2: Cos date	0.235	0.133	-0.020	0.501
Time of day: Cos date	-0.035	0.065	-0.164	0.094
Time of day^2 : Sex ratio	-0.217	0.214	-0.647	0.195
Time of day : Sex ratio	0.116	0.104	-0.089	0.317
Time of day^2 : Community size	-0.160	0.275	-0.699	0.376
Time of day : Community size	0.163	0.127	-0.078	0.410
Time of day^2 : LCMS code (new method)	-0.419	0.366	-1.102	0.312
Time of day : LCMS code (new method)	0.349	0.196	-0.039	0.723

*Table S9: Random effect results from the reaction norm LMM estimating urinary cortisol concentrations (ng/ml SG) in response to time of day among **immatures** in five chimpanzee communities.*

	Name	Variance	Est.error	Q2.5	Q97.5
Group		0.73	0.65	0.05	2.46
Project identity		0.39	0.14	0.18	0.72
Group-year		0.30	0.13	0.03	0.56
ID-year	Intercept	0.32	0.08	0.15	0.47
	Time of day^2	0.59	0.18	0.14	0.90
	Time of day	0.26	0.09	0.06	0.42
Individual identity	Intercept	0.27	0.09	0.07	0.43
	Time of day^2	0.22	0.16	0.01	0.58
	Time of day	0.13	0.08	0.01	0.31
Residual		0.58	0.01	0.56	0.61

Table S10: Medians of MCMC iterations (median) and their 5% and 95% quantiles for random effects included in the animal model and heritability calculations. Note that the technical predictor, project identity, is excluded to compute the proportion of variance in inter-individual differences. The medians for all predictors combined are provided in Table S4.

**Predictor**	**Coefficient**	**Median proportion of variance**	**Lower CI**	**Upper CI**
Genetic	Intercept	0.049	0.004	0.136
	Time of day	0.029	0.002	0.084
	Time of day^2	0.055	0.005	0.162
	Covariance (Intercept, Time of day)	0.111	-0.768	0.841
	Covariance (Intercept, Time of day^2)	-0.119	-0.842	0.764
	Covariance (Time of day, Time of day^2)	-0.045	-0.827	0.784
Maternal identity	Intercept	0.185	0.084	0.245
	Time of day	0.049	0.004	0.096
	Time of day^2	0.053	0.005	0.156
	Covariance (Intercept, Time of day)	0.301	0.604	0.856
	Covariance (Intercept, Time of day^2)	-0.206	-0.847	0.720
	Covariance (Time of day, Time of day^2)	-0.028	-0.815	0.0795
Individual identity	Intercept	0.078	0.008	0.188
	Time of day	0.031	0.003	0.086
	Time of day^2	0.061	0.006	0.174
	Covariance (Intercept, Time of day)	0.095	-0.766	0.835
	Covariance (Intercept, Time of day^2)	-0.118	-0.834	0.757
	Covariance (Time of day, Time of day^2)	-0.062	-0.830	0.782
ID-year	Intercept	0.223	0.184	0.267
	Time of day	0.055	0.005	0.134
	Time of day^2	0.179	0.022	0.337

	Covariance (Intercept, Time of day)	-0.137	-0.763	0.604
	Covariance (Intercept, Time of day^2)	-0.330	-0.742	0.416
	Covariance (Time of day, Time of day^2)	-0.146	-0.842	0.740
Group-year	Intercept	0.583	0.452	0.747
	Time of day	0.080	0.011	0.173
	Time of day^2	0.153	0.019	0.342
	Covariance (Intercept, Time of day)	0.105	-0.672	0.748
	Covariance (Intercept, Time of day^2)	-0.750	-0.971	0.248
	Covariance (Time of day, Time of day^2)	-0.121	-0.807	0.717
Database-code	Intercept	0.584	0.306	1.071
	Time of day	0.210	0.106	0.384
	Time of day^2	0.164	0.019	0.395
	Covariance (Intercept, Time of day)	-0.668	-0.924	-0.037
	Covariance (Intercept, Time of day^2)	0.074	-0.705	0.791
	Covariance (Time of day, Time of day^2)	-0.094	-0.773	0.693
Residual error		0.650	0.639	0.660

*Table S11. Summary of genetic and maternal effect estimates on circadian cortisol responses in wild chimpanzees when only samples from **Taï groups** were used. Each coefficient represents a different component of the cortisol response. We also report the proportion of permutations for which these coefficient estimates were less than in the observed data. Coefficients in bold were larger in our observed data than in at least 95% of our random permutations. The estimated effect (estimate) is reported in terms of proportion of explained within-group variance for within-group effects, and total variance for between-group effects.*

**Coefficient**	**Estimate**	**(lCI, uCI)**	**Proportion observed < permutations**
	**Within group effects**		
Genetic effect			
Intercept	0.009	(0.000, 0.074)	0.76
Linear	0.067	(0.001, 0.495)	0.87
Quadratic	0.031	(0.000, 0.279)	0.83
Maternal effect			
**Intercept**	**0.082**	**(0.009, 0.174)**	**0.02**
**Linear**	**0.127**	**(0.001, 0.639)**	**0.03**
**Quadratic**	**0.024**	**(0.000, 0.250)**	**0.12**
Group-year effect			
Intercept	0.772	(0.639, 0.863)	/
Linear	0. 266	(0.004, 0.801)	/
Quadratic	0. 554	(0.024, 0.911)	/
ID-year effect			
Intercept	0.095	(0.049, 0.175)	/
Linear	0.128	(0.001, 0.674)	/
Quadratic	0.222	(0.003, 0.763)	/
Individual identity			
Intercept	0.015	(0.000, 0.099)	/
Linear	0.073	(0.001, 0.522)	/
Quadratic	0.033	(0.000, 0.302)	/
	**Between group effects**		
Group/Shared communal effects			
Intercept	0.980	(0.930, 0.995)	/
Linear	0.636	(0.010, 0.990)	/
Quadratic	0.257	(0.000, 0.931)	/

*Table S12. Summary of genetic and maternal effect estimates on circadian cortisol responses in wild chimpanzees including individuals to which we could assign **dominance rank**. Each coefficient represents a different component of the cortisol response. We also report the proportion of permutations for which these coefficient estimates were less than in the observed data. Coefficients in bold were larger in our observed data than in at least 95% of our random permutations. The estimated effect (estimate) is reported in terms of proportion of explained within-group variance for within-group effects, and total variance for between-group effects.*

Coefficient	Estimate	(lCI, uCI)	Proportion observed < permutations
Genetic effect			
Intercept	0.006	(0,0.050)	0.91
Linear	0.059	(0.001,0.452)	0.87
Quadratic	0.034	(0,0.322)	0.82
Maternal effect			
**Intercept**	**0.054**	**(0.003,0.130)**	**0.04**
Linear	0.086	(0.001, 0.546)	0.21
**Quadratic**	**0.032**	**(0.000, 0.295)**	**0.02**
Group-year effect			
Intercept	0.773	(0.661,0.859)	/
Linear	0.317	(0.004,0.818)	/
Quadratic	0.280	(0.005,0.809)	/
ID-year effect			
Intercept	0.119	(0.058,0.182)	/
Linear	0.149	(0.001, 0.707)	/
Quadratic	0.422	(0.011, 0. 869)	/
Individual identity			
Intercept	0.024	(0.000,0.107)	/
Linear	0.044	(0.000, 0.367)	/
Quadratic	0.080	(0.001, 0.532)	/
**Between group effects**			
Group/Shared communal effects			
Intercept	0.976	(0.916, 0.993)	/
Linear	0.527	(0.006, 0.986)	/
Quadratic	0.212	(0.015, 0.902)	/

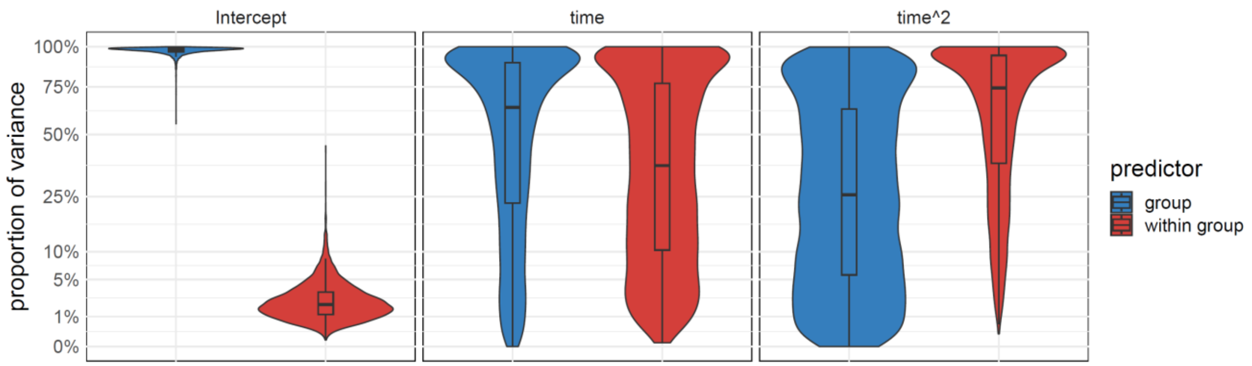

Figure S6: Estimates for the proportion of variance in cortisol in wild chimpanzees explained by random effects for a model in which only samples from the Taï population are used (Taï model). The posterior distribution of the proportion of explained variance is shown as violin plots, with interquartile ranges represented by boxplots.

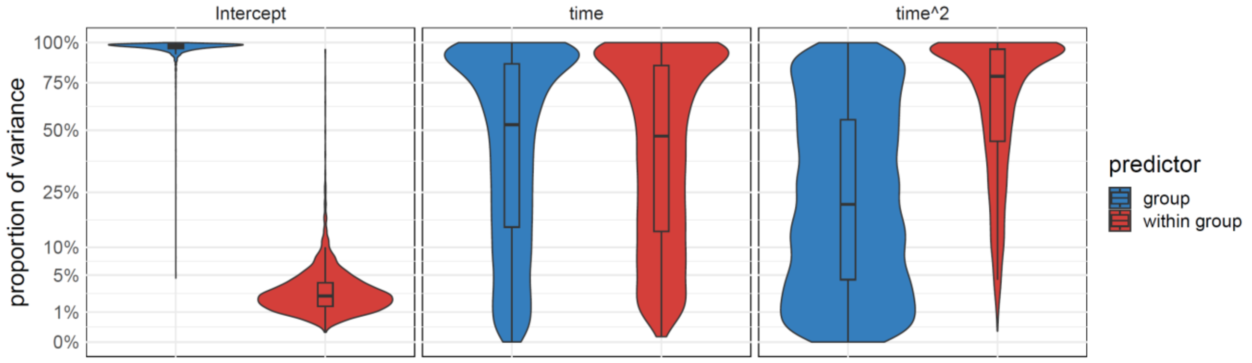

Figure S7: Estimates for the proportion of variance in cortisol in wild chimpanzees explained by random effects for the Dominance heritability models. The posterior distribution of the proportion of explained variance is shown as violin plots, with interquartile ranges represented by boxplots.

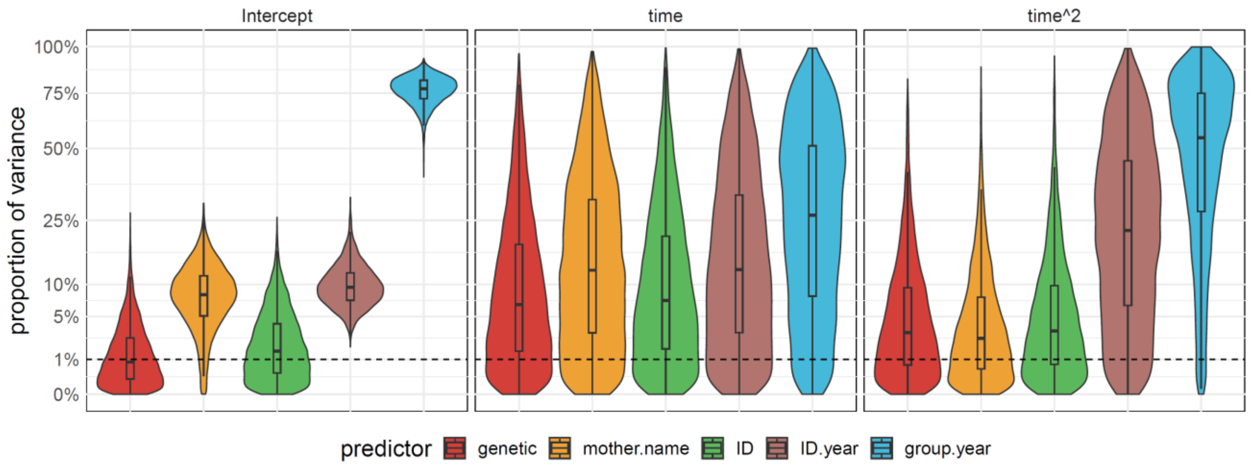

Figure S8: Estimates for the proportion of within-group variance in cortisol in wild chimpanzees explained by random effects for a model in which only samples from the Taï population are used (Taï model). The posterior distribution of the proportion of explained variance is shown as violin plots, with interquartile ranges represented by boxplots. The horizontal dashed line marks a proportion of within-group variance of 1%.

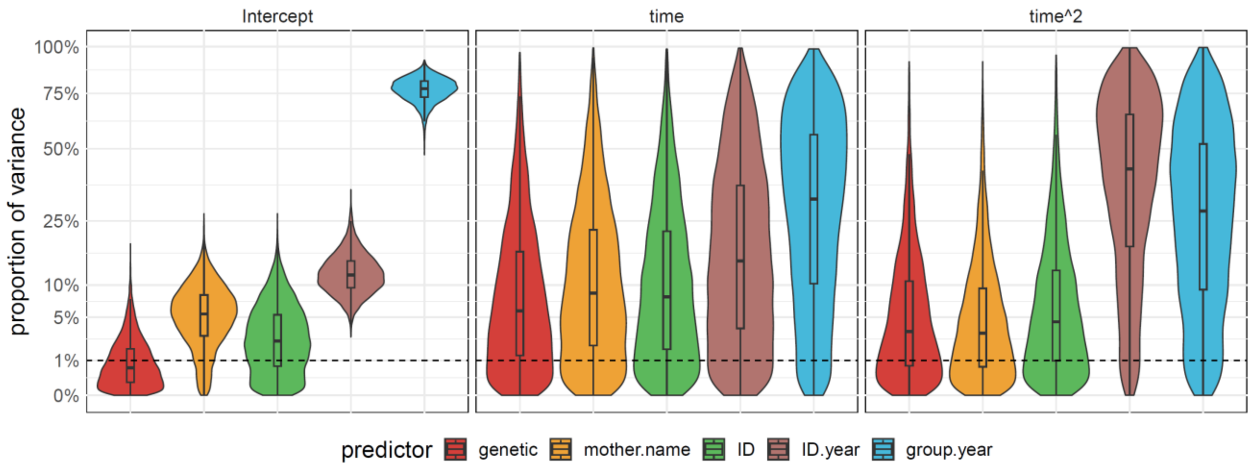

Figure S9: Estimates for the proportion of within-group variance in cortisol in wild chimpanzees explained by random effects for a model in which only samples with rank information are used, and rank is used as a fixed predictor (Dominance heritability model). The posterior distribution of the proportion of explained variance is shown as violin plots, with interquartile ranges represented by boxplots. The horizontal dashed line marks a proportion of within-group variance of 1%.

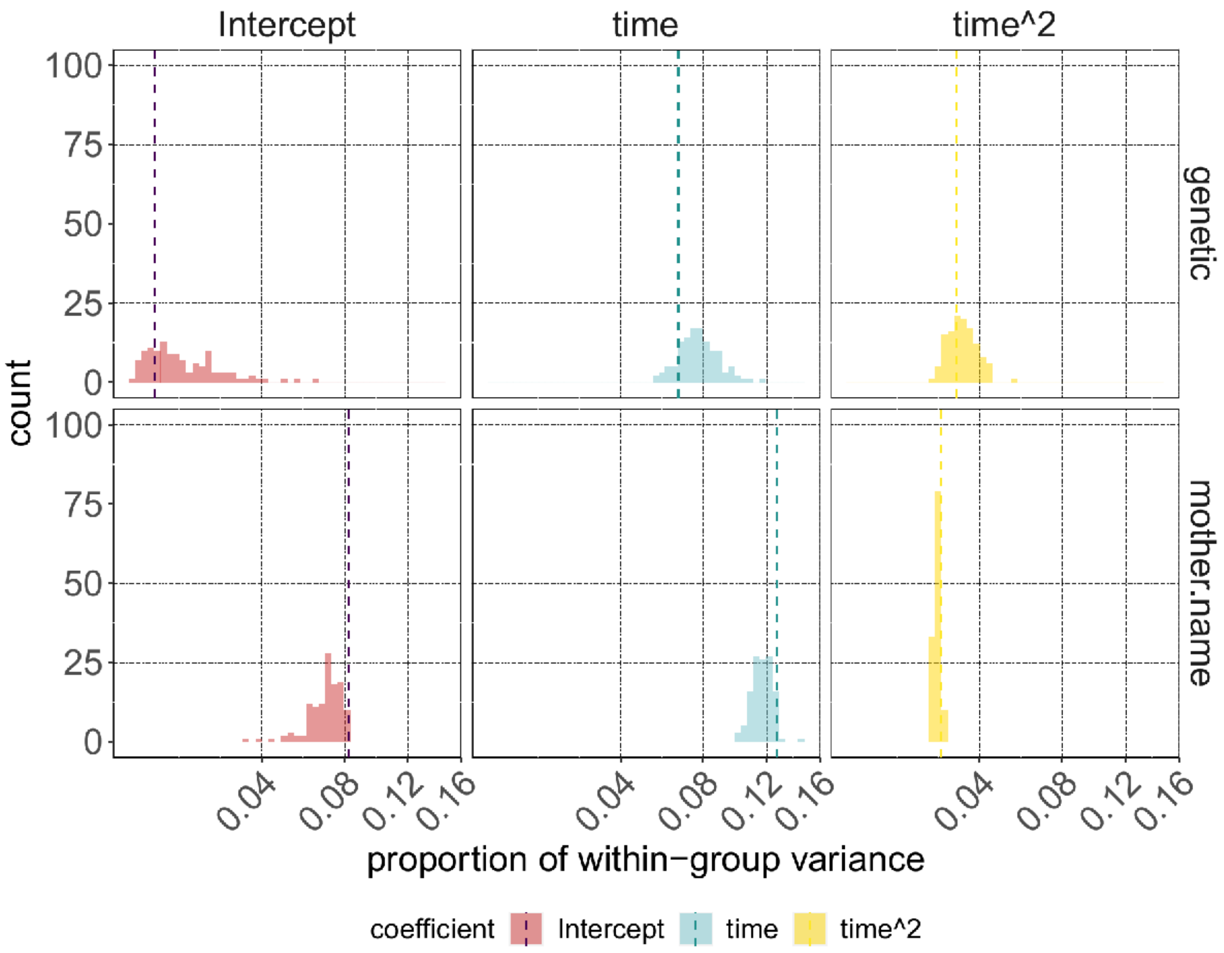

Figure S10: Comparison of genetic and maternal within-group variance estimates obtained from observed versus permuted data for the Taï model. Median proportion of variance estimates obtained from the observed data are represented by dashed vertical lines; histograms represent the counts of each estimate value from 100 permutations.

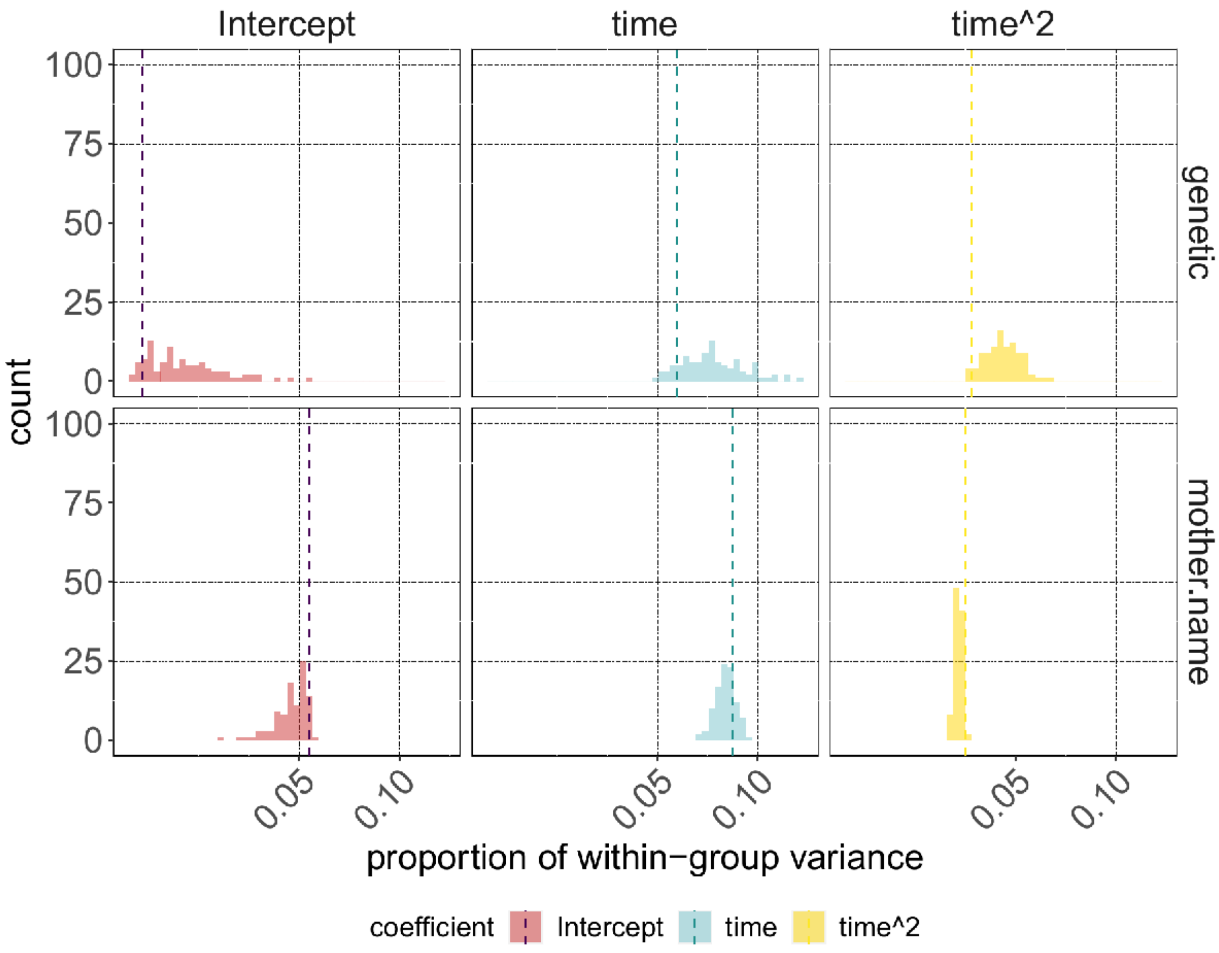

Figure S11: Comparison of genetic and maternal within-group variance estimates obtained from observed versus permuted data for the Dominance heritability model. Median proportion of variance estimates obtained from the observed data are represented by dashed vertical lines; histograms represent the counts of each estimate value from 100 permutations.

Table S13. Summary of genetic and maternal effect estimates on cortisol responses in wild chimpanzees in the Trait heritability model. We also report the proportion of permutations for which these coefficient estimates were less than in the observed data. Coefficients in bold were larger in our observed data than in at least 95% of our random permutations. The estimated effect (estimate) is reported in terms of proportion of explained within-group variance for within-group effects, and total variance for between-group effects and residual variance.

Coefficient	Estimate	(lCI, uCI)	Proportion observed < permutations
**Within group effects**			
Genetic effect	0.007	(0,0.024)	0.95
Maternal effect	**0.095**	**(0.013,0.176)**	**0.00**
Group-year effect	**0.725**	**(0.611,0.820)**	/
ID-year effect	**0.134**	**(0.082,0.207)**	/
Individual identity	0.018	(0,0.109)	/
**Between group effects**			
**Group/shared communal effects**	**0.976**	**(0.916, 0.993)**	**/**
Residual	0.527	(0.006, 0.986)	/

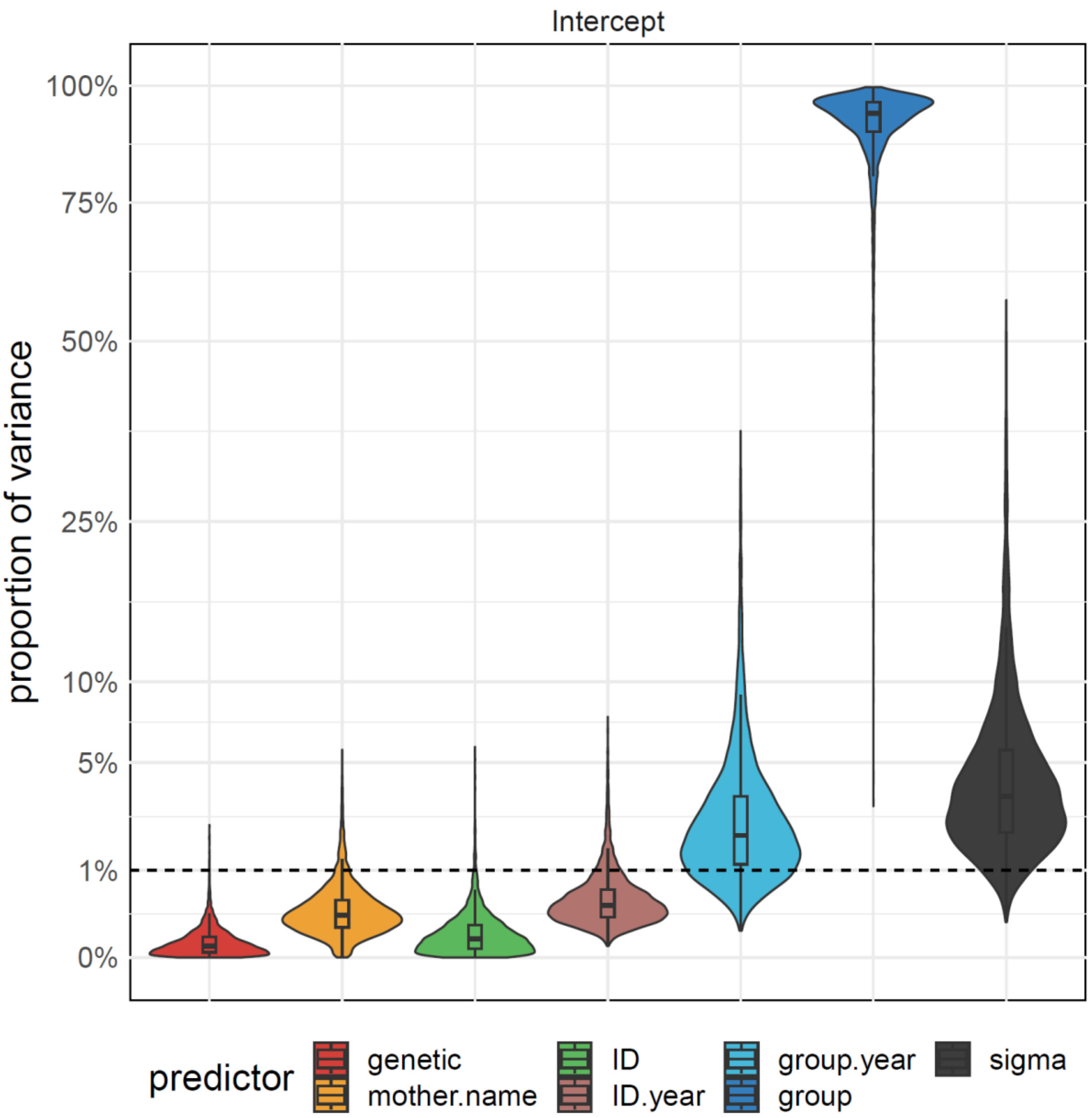

Figure S12: Estimates for the proportion of variance in cortisol in wild chimpanzees for the Trait heritability model. Sigma stands for the residual variance. The posterior distribution of the proportion of explained variance is shown as violin plots, with interquartile ranges represented by boxplots. The horizontal dashed line marks a proportion of within-group variance of 1%.

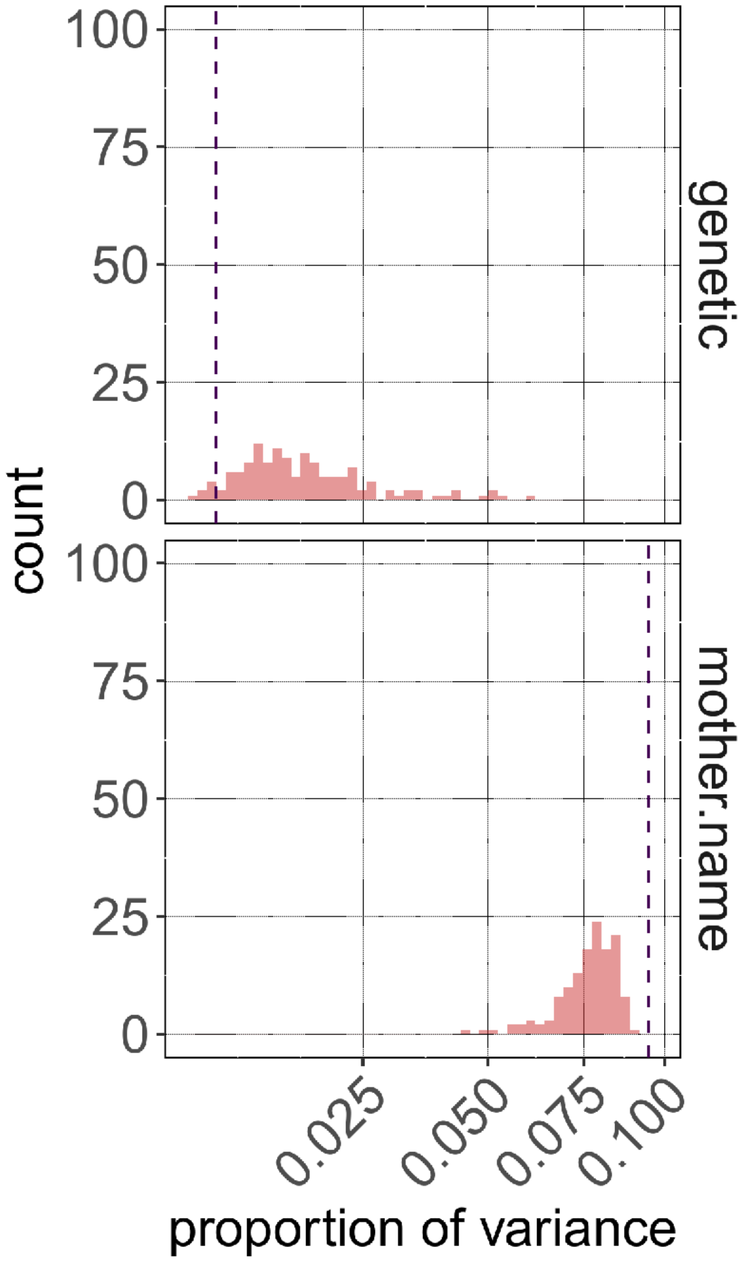

Figure S13: Comparison of genetic and maternal within-group variance estimates obtained from observed versus permuted data for the Trait heritability model. Median proportion of variance estimates obtained from the observed data are represented by dashed vertical lines; histograms represent the counts of each estimate value from 100 permutations.

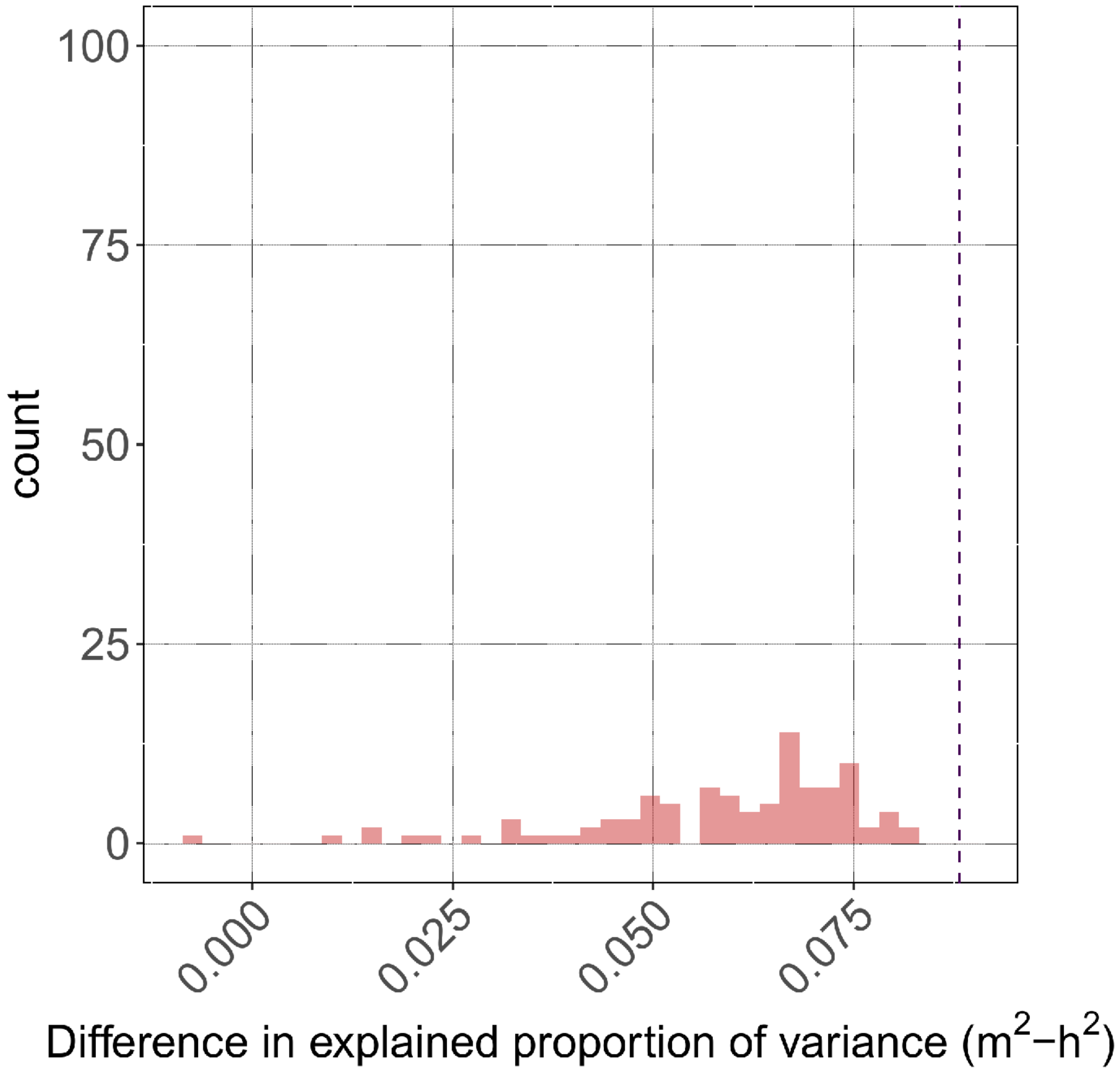

Figure S14: Estimates of the difference in the proportion of within-group variance explained by the maternal effect and that explained by genetic factors in the observed data (dashed line) and in 100 permutations of the data (histogram) in the Trait heritability model.

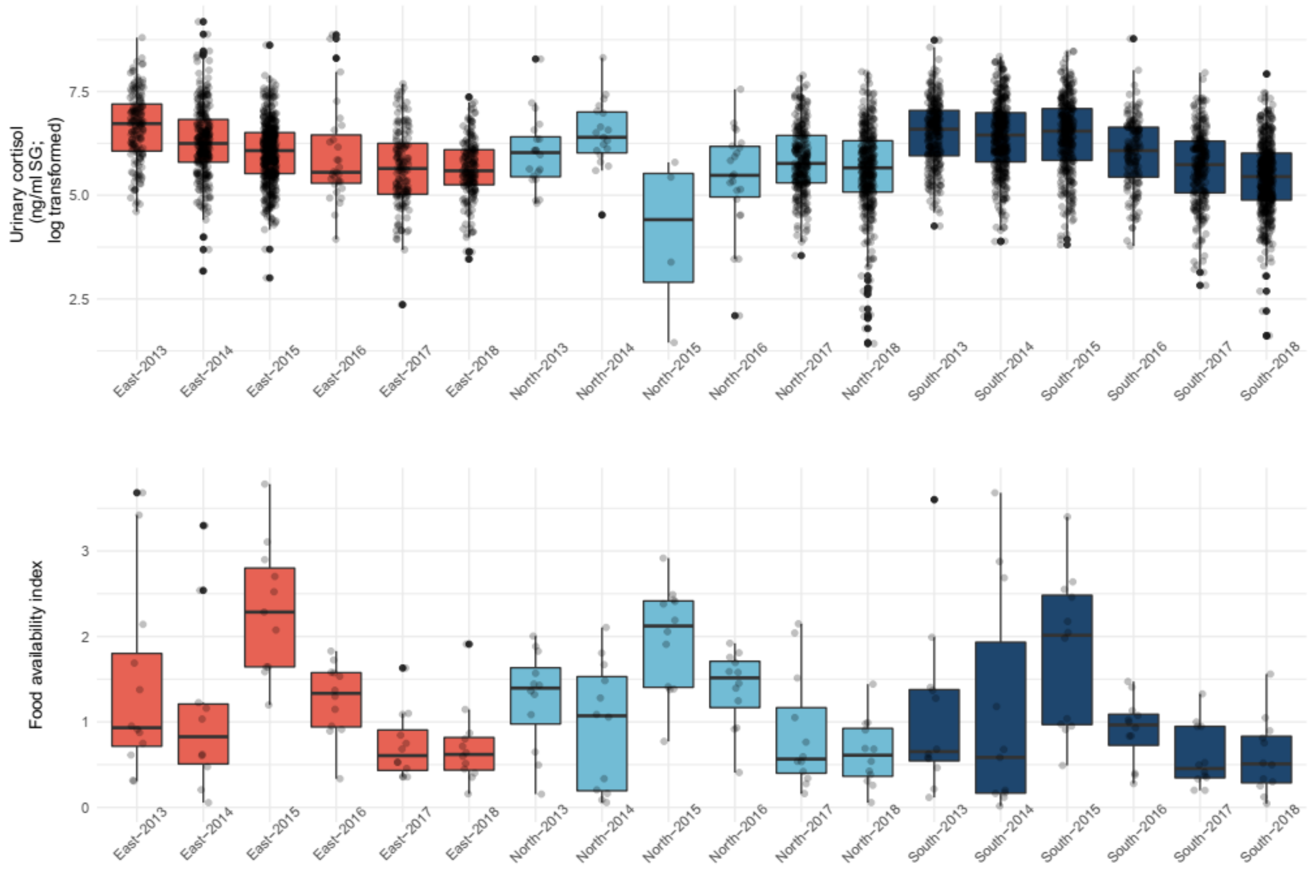

Figure S15: Group level differences in urinary cortisol levels and food availability in Taï. All group-years in which we had data for each of these variables are presented (i.e., between 2013-2018). In the upper half of the plot, each point represents urinary cortisol concentration (ng/mL SG, log transformed; n=3,977); in the lower plot each point represents a monthly food availability index value (n=212). In upper and lower plots, the boxes represent the interquartile range, median, minimum and maximum range of urinary cortisol concentrations and food availability indices respectively. The food availability index was calculated using the mean basal area of tree species (phenology data), the percentage of observed fruiting tree species, and the density of tree species (see Valé et al, 2020; (69) in references of main manuscript).

Table S14. Summary of variance components from models run to test potential temporal batch effects on our main model estimates. A model including all samples was identical to our main heritability model but included an additional random effect of month-year; a restricted model was also run, which included the month-year effect but limiting to analysis to data points for which data were available for more than one population in any month-year time period.

**Coefficient**	**All – Prop variance median**	**(lCI, uCI)**	**Restricted – Prop variance median**	**(lCI, uCI)**
Genetic effect				
Intercept	0.000	(0.000, 0.003)	0.000	(0.000, 0.003)
Linear	0.012	(0.000, 0.107)	0.018	(0.000, 0.166)
Quadratic	0.011	(0.000, 0.131)	0.008	(0.000, 0.114)
Maternal effect				
Intercept	0.002	(0.000, 0.011)	0.002	(0.000, 0.011)
Linear	0.011	(0.000, 0.102)	0.016	(0.000, 0.158)
Quadratic	0.025	(0.000, 0.196)	0.017	(0.000, 0.164)
Group-year effect				
Intercept	0.021	(0.006, 0.088)	0.014	(0.003, 0.070)
Linear	0.150	(0.005, 0.482)	0.042	(0.000, 0.346)
Quadratic	0.041	(0.000, 0.326)	0.040	(0.000, 0.331)
ID-year effect				
Intercept	0.004	(0.001, 0.018)	0.005	(0.001, 0.020)
Linear	0.084	(0.001, 0.373)	0.058	(0.001, 0.347)
Quadratic	0.030	(0.000, 0.277)	0.029	(0.000, 0.285)
Individual identity				
Intercept	0.000	(0.000, 0.005)	0.001	(0.000, 0.006)
Linear	0.014	(0.000, 0.129)	0.024	(0.000, 0.201)
Quadratic	0.012	(0.000, 0.139)	0.010	(0.000, 0.125)
Group/Shared communal effects				
Intercept	0.967	(0.868, 0.991)	0.974	(0.880, 0.993)
Linear	0.090	(0.001, 0.717)	0.161	(0.002, 0.806)
Quadratic	0.322	(0.002, 0.946)	0.441	(0.005, 0.952)
Month-year effect				
Intercept	0.003	(0.001, 0.014)	0.003	(0.001, 0.015)
Linear	0.412	(0.112, 0.748)	0.410	(0.088, 0.761)
Quadratic	0.323	(0.027, 0.715)	0.260	(0.023, 0.679)

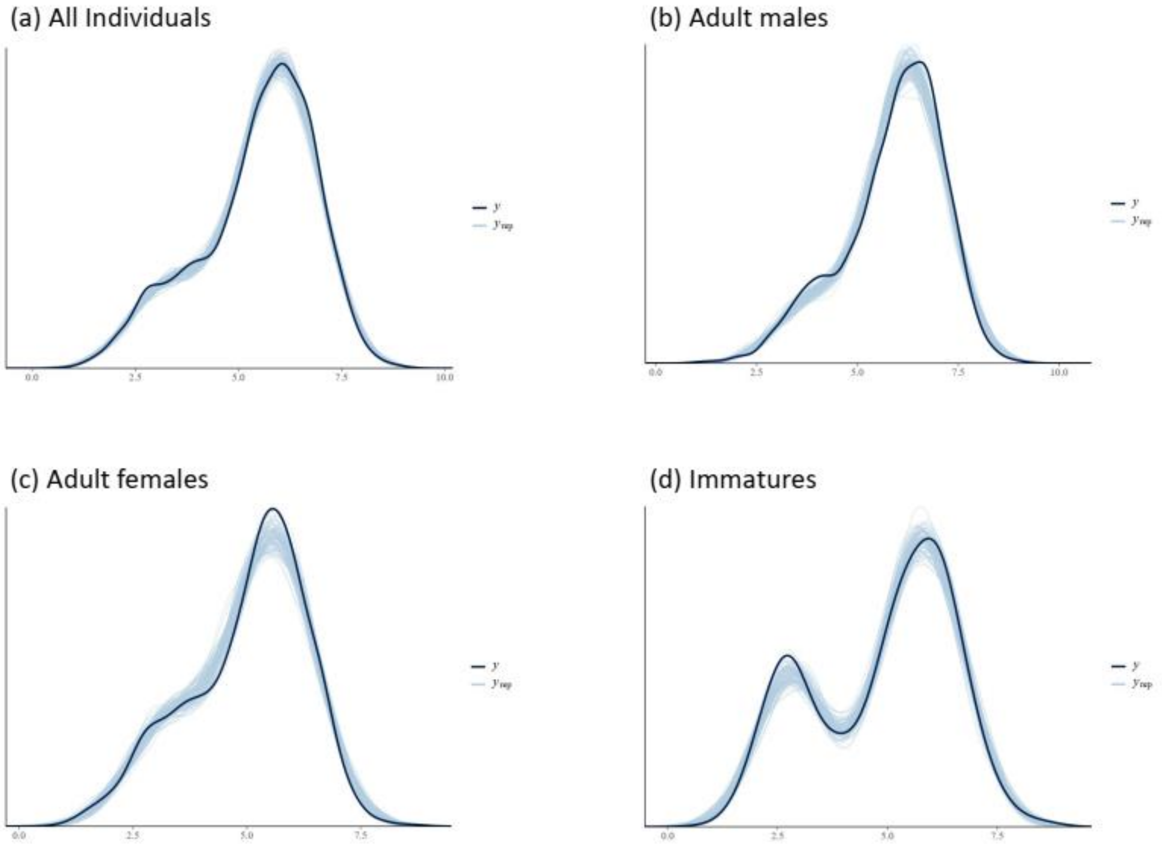

Figure S16: Posterior predictive checks for the (a) all individuals, (b) adult male, (c) adult female, and (d) immature reaction norm models used for the repeatability analyses. Light blue lines indicate 100 draws from the posterior, the solid blue line represents the observed data.

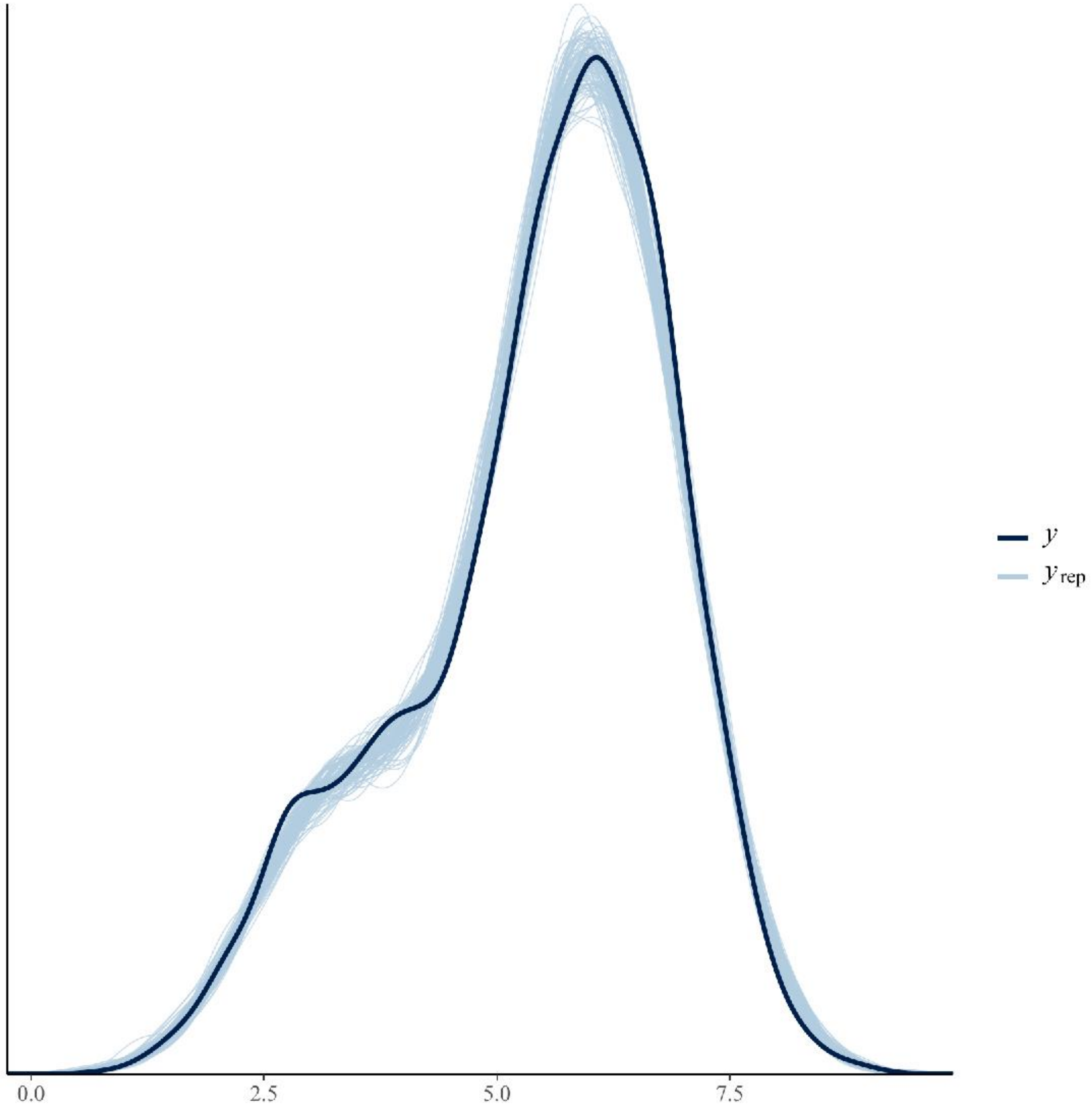

Figure S17: Posterior predictive checks for the Full heritability model. Light blue lines indicate 100 draws from the posterior, the solid blue line represents the observed data.
